# Appendicular Band Syndrome simulating Appendicular Mass in a Child

**Published:** 2014-09-01

**Authors:** Bilal Mirza, Muhammad Saleem

**Affiliations:** Department of Pediatric Surgery, The Children’s Hospital and the Institute of Child Health Lahore

**Keywords:** Appendicular Band, Knot, Appendicular Mass, Strangulation

## Abstract

Appendicular band syndrome is an exceedingly rare surgical emergency that may lead to intestinal obstruction and strangulation. We report a case of 2-year-old boy who presented with acute intestinal obstruction with a mass in right iliac fossa (RIF). At exploration, an inflamed appendix had entrapped a loop of terminal ileum leading to its strangulation and gangrene. The appendectomy and resection of gangrenous gut were done with formation of an ileostomy.

## INTRODUCTION

Appendicular band syndrome also called as appendicular knot or appendicular tie syndrome is an extremely rare surgical entity. About 11 cases are reported so far.[1-5] It usually presents with intestinal obstruction. The gut entrapped by the appendix, acting as constricting band, may strangulate leading to gangrene, if not intervened early.[3,5] We herein report a case of appendicular band syndrome who presented with signs of intestinal obstruction and a palpable mass in RIF that simulated appendicular mass.

## CASE REPORT

A 2-year-old boy presented with abdominal distension, constipation, and bilious vomiting for three days. There was a history of irritability and fever a week before presenting to us. On examination, the patient was febrile (temp, 100°F) with tachycardia (pulse 140/min) and dehydrated. Abdomen was distended with visible bowel loops. A mass was palpable in the RIF with tenderness and guarding in the lower abdomen. A digital rectal examination yielded empty rectum. X-ray abdomen erect showed a complete cut-off sign indicating intestinal obstruction. Ultrasound abdomen depicted excessive gaseous shadows in the lower abdomen. Laboratory test showed Hb 10g/dl and WBC count of 22000 with 80% of neutrophils. A diagnosis of appendicular mass/abscess with bowel obstruction was made. The patient was optimized for operation. At operation, the vermiform appendix was found encircling a loop of terminal ileum like a band. The tip of appendix was adherent with the cecum; the entrapped ileum was gangrenous (Fig. 1, 2). The tip of ileum which was inflamed freed from the cecum and appendectomy performed. The gangrenous ileum was resected and an ileostomy formed 5cm proximal to ileocecal valve. The postoperative recovery was uneventful. The patient is on our follow-up for ileostomy reversal.

**Figure:**
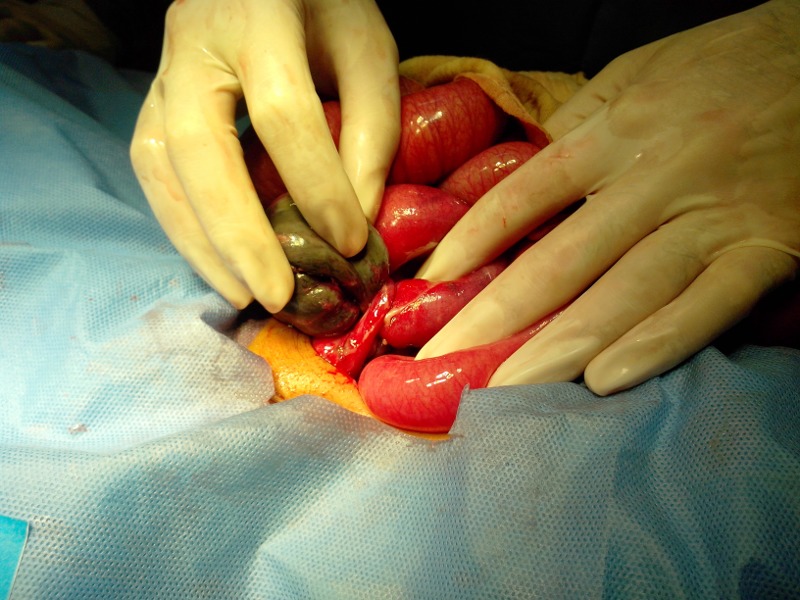
Figure 1:Appendicular band around terminal ileum causing bowel necrosis.

**Figure:**
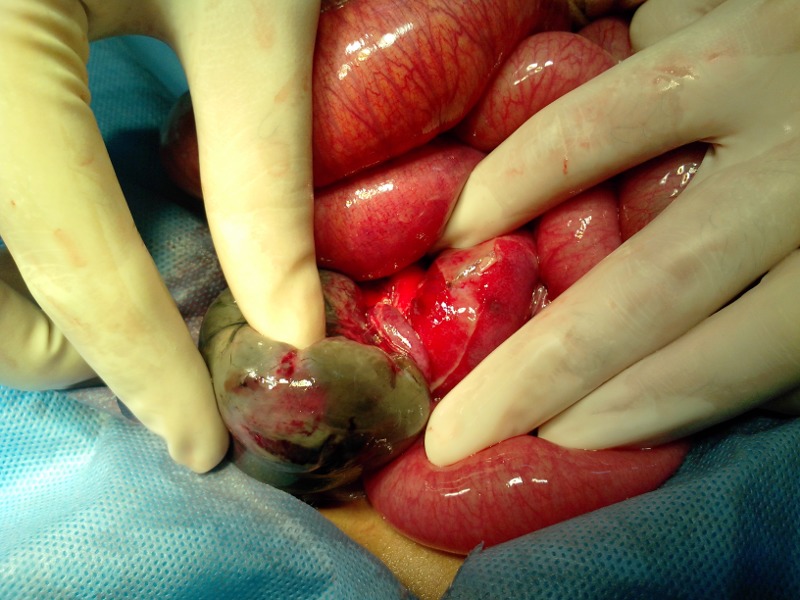
Figure 2:Another view

## DISCUSSION

Appendicular band syndrome is reported in neonate, children, as well as adults.[1-5]It is not clear how the appendix entraps forming a band or tie. The tip of a long appendix is usually found adhered to cecum, retroperitoneum, mesentery of ileum, or rarely ileum itself thus forming a potential space where a loop of bowel may entrap.[3] Acute inflammation of the appendix is probably the inciting event of this band formation. The appendix itself may be acutely inflamed, perforated especially at the tip or it may be completely gangrenous. This entrapment not only results in intestinal obstruction and strangulation of the entrapped bowel but it can also result in ischemia of appendix itself due to compression. Chintamani et al [6] reported a case where gangrenous appendix was forming a knot around a loop of ileum. In their case the entrapment might have resulted in compression-strangulation of the appendix as otherwise the appendix must be extensively inflamed if appendicitis had led to the gangrenous appendix.

The reported complications are intestinal obstruction, volvulus, strangulation of the small bowel, and strangulation of appendix itself.[1-6] In our case the presentation was also with intestinal obstruction but a mass in the RIF simulated an appendicular mass. The gangrenous, entrapped, distended, and tense loop of ileum was palpable as a mass. In majority of the cases this entity is diagnosed at operation. If the entrapped bowel is not compromised, appendectomy is the only treatment required for unraveling the tie.[2,6] In case of compromised loop [3] as in the index case resection is needed.

To summarize, appendicular tie syndrome is an extremely rare surgical emergency which may lead to bowel necrosis if not promptly treated.

## Footnotes

**Source of Support:** Nil

**Conflict of Interest:** None declared

